# Gamma-glutamyl transferase and pulmonary embolism: Gama glutamil transferase e embolia pulmonar

**DOI:** 10.1590/1516-3180.2016.0080080516

**Published:** 2016-09-19

**Authors:** Sevket Balta, Cengiz Ozturk, Mustafa Demir, Ali Osman Yildirim, Turgay Celık

**Affiliations:** I MD. Staff Cardiologist, Department of Cardiology, Gulhane Medical Academy, Ankara, Turkey.; II MD. Associate Professor of Cardiology, Department of Cardiology, Gulhane Medical Academy, Ankara, Turkey.; III MD. Resident of Cardiology, Department of Cardiology, Gulhane Medical Academy, Ankara, Turkey; IV MD. Resident of Cardiology, Department of Cardiology, Gulhane Medical Academy, Ankara, Turkey.; V MD. Professor of Cardiology, Department of Cardiology, Gulhane Medical Academy, Ankara, Turkey.

Dear Editor,

We read the article “Elevated gamma glutamyl transferase (GGT) levels are associated with the location of acute pulmonary embolism. Cross-sectional evaluation in hospital setting” by Korkmaz et al.^1^ They concluded that there was a significant association between increased embolism load in the pulmonary artery and increased serum GGT levels.

Several noninvasive tests have been used for accurately diagnosing pulmonary embolism. Some inflammatory markers like red cell distribution width may show pulmonary embolism through further diagnostic tests.^2^ In addition, mean platelet volume may describe or predict pulmonary embolism.^3^ Tests for these markers are routine, available and accessible through any laboratory. Serum GGT activity is a marker of oxidative stress and endothelial dysfunction. We previously showed that serum GGT level may be an independent marker for the severity of cardiovascular diseases.^4^ Korkmaz et al.^1^ showed the correlation between GGT and acute pulmonary embolism. However, some important issues need to be considered. Firstly, GGT determination is one of the hepatic function tests. Presence of GGT may change any form of hepatic dysfunction even if no overt liver disease is present. Secondly, Gilbert syndrome is frequently seen worldwide. This syndrome may be correlated with hepatic function tests. GGT is also a widely measured serum enzyme: it almost specifically shows biliary epithelial damage and generally indicates excessive alcohol intake. GGT is released from many tissues such as the liver, kidney, cerebrovascular endothelium and pericytes.^5^ Furthermore, changes in serum GGT levels can be affected by waist circumference and body mass index, hypertension, diabetes, hyperuricemia and genetic factors. Lastly, admission troponin levels were assessed in the recent study by Korkmaz et al.,^1^ but the patients seemed to have a relatively long duration of symptoms before admission, which may have led to underestimation of the potential impact of troponin.

In conclusion, although GGT levels were associated with pulmonary embolism in the study published in this journal,^1^ GGT levels alone may not provide enough information to diagnose these patients, unless the factors outlined above are also taken into consideration. Therefore, we believe that GGT should be evaluated under these conditions. We believe that these findings will be clarified through further studies.
